# Polycythemia Vera Transforming to Pure Erythroid Leukemia: A Diagnostic Mimic of Thrombotic Microangiopathy With High‐Risk TP53 Biology

**DOI:** 10.1155/crh/8549290

**Published:** 2026-10-15

**Authors:** Thalia Padilla Kelley, Lam Duc, Jean Sabile, Ronan Swords

**Affiliations:** ^1^ Department of Medicine Oregon Health & Science University Portland Oregon USA ohsu.edu; ^2^ Division of Hematology and Medical Oncology Oregon Health & Science University Portland Oregon USA ohsu.edu

**Keywords:** acute myeloid leukemia, bone marrow biopsy, JAK2 V617F, leukemic transformation, myeloproliferative neoplasm, polycythemia vera, pure erythroid leukemia, thrombotic microangiopathy, thrombotic thrombocytopenic purpura, TP53 mutation

## Abstract

Leukemic transformation of polycythemia vera (PV) to acute myeloid leukemia (AML) with a predominantly erythroid blast population is rare and carries a poor prognosis, particularly when driven by a TP53 mutation. We report a 55‐year‐old man with JAK2 V617F‐positive PV, previously noted on surveillance biopsy to harbor a TP53 mutation and Grade 2 marrow fibrosis, who presented three years later with pancytopenia, schistocytosis, hyperferritinemia, hypofibrinogenemia, and altered mental status. The initial clinical presentation raised concern for thrombotic thrombocytopenic purpura (TTP), while the presence of rare circulating blasts and coagulopathy also prompted consideration of acute promyelocytic leukemia (APML). Empiric plasma exchange, corticosteroids, and all‐trans retinoic acid were initiated. ADAMTS13 activity and PML‐RARA FISH subsequently returned negative. The patient deteriorated with multiorgan failure and expired before leukemia‐directed therapy could be initiated. Postmortem bone marrow results demonstrated greater than 90% erythroid blasts with TP53 protein overexpression, a TP53 p.C238Y mutation (VAF 66%), and a triploid karyotype with 7p and 19p abnormalities, consistent with pure erythroid leukemia (PEL) arising from an antecedent myeloproliferative neoplasm. This case highlights the diagnostic challenges posed by overlapping clinical features of TTP and APML, including limitations in immunophenotypic assignment of the erythroid lineage. Importantly, it provides a longitudinally documented example of a TP53 mutation detected three years before overt transformation and describes a previously unreported presentation mimicking both TTP and APML.

## 1. Introduction

Acute myeloid leukemia (AML) with a predominant exclusive erythroid blast population is a rare and biologically aggressive presentation, accounting for fewer than 1% of AML diagnoses, and carries a uniformly poor prognosis [1, 2]. Terminology and diagnostic criteria for this presentation differ between the two major contemporary classification systems. The 2022 International Consensus Classification (ICC) of myeloid neoplasms recognizes AML with TP53 mutation as a distinct entity, encompassing cases that were previously classified as pure erythroid leukemia (PEL), whereas PEL is not retained as a distinct entity in this classification. The World Health Organization Classification of Haematolymphoid Tumours (WHO‐HAEM5), in contrast, retains PEL as a distinct entity, defined by predominant erythroid differentiation with proerythroblasts comprising ≥ 30% of bone marrow cells and erythroid precursors comprising ≥ 80% of nucleated cells [3, 4]. Thus, cases with erythroid predominance and TP53 mutation may be classified as AML with TP53 mutation under the ICC but as PEL under the WHO‐HAEM5. This distinction is particularly relevant in the present case, as the patient’s marrow findings and TP53 mutation result in different diagnostic classifications depending on the system applied. These differences have important implications for interpreting the diagnosis and comparing the case with previously reported PEL cases.

PEL most commonly arises as a secondary transformation from antecedent hematologic conditions, particularly myelodysplastic syndromes (MDSs) and myeloproliferative neoplasms (MPNs), and infrequently emerges as a de novo malignancy [1, 2]. Among the MPNs, polycythemia vera (PV) is a recognized, albeit uncommon, antecedent to leukemic transformation. The risk of progression from PV to AML has been estimated at approximately 2.3% over 10 years, rising to as high as 18% over 15 years, with transformation driven by the sequential acquisition of somatic mutations beyond the initiating JAK2 V617F alteration [5, 6]. Mutations in TP53 are disproportionately represented in erythroid‐predominant AML and in MPN‐derived AML more broadly and are associated with complex or nondiploid karyotypes, marked resistance to conventional cytotoxic therapy, and inferior overall survival [7, 8]. Prior studies have established that JAK2/TP53 comutated AML with erythroid or megakaryocytic differentiation represents a high‐risk form of TP53‐mutated AML, with experimental data supporting a role for these mutations in erythroid/megakaryocytic‐skewed leukemogenesis [9]. Our case adds to this literature by illustrating the clinical and diagnostic challenges of PV‐to‐AML transformation, which initially mimicked TMA and raised concern for APML, while demonstrating a marrow phenotype that may be classified differently across contemporary diagnostic frameworks.

## 2. Case Presentation

A 55‐year‐old Filipino man with a history of JAK2 V617F‐positive PV presented to an outside emergency department with a two‐week history of progressive exertional dyspnea, profound generalized weakness, and bilateral lower extremity edema. He additionally reported a one‐month history of reduced urinary output. His past medical history was significant for PV diagnosed in 2018 following an acute ischemic cerebrovascular accident attributed to extreme erythrocytosis; at that time, his hemoglobin measured 21.4 g/dL and hematocrit 69.7%. He was subsequently managed with hydroxyurea (1500 mg on weekdays and 1000 mg on weekends), aspirin 162 mg twice daily, and intermittent weekly therapeutic phlebotomy, with initial hematologic stability.

In February 2022, a surveillance bone marrow biopsy demonstrated markedly hypercellular marrow (80%–85% cellularity) with myeloid and megakaryocytic hyperplasia and increased reticulin fibrosis graded MF‐2 by the European Consensus grading system [10]. Concurrent molecular profiling identified a JAK2 V617F mutation (variant allele fraction [VAF] 70.7%) and a TP53 mutation (VAF 33.1%). Flow cytometry at that time revealed a population of CD34‐positive myeloblasts with additional basophilic and monocytic populations exhibiting mild, nonspecific immunophenotypic aberrancies of uncertain clinical significance. The patient was followed closely in the outpatient hematology clinic with continuation of hydroxyurea and ongoing phlebotomy.

### 2.1. Initial Presentation and Laboratory Findings

At the time of initial evaluation at the outside institution, laboratory studies demonstrated new‐onset pancytopenia, with a white blood cell (WBC) count of 2.7 × 10^3^ cells/uL, hemoglobin of 11.8 g/dL, and platelet count of 37 × 10^3^ cells/uL. A mild acute kidney injury was also noted, with a serum creatinine of 1.23 mg/dL, along with hyponatremia (sodium 131 mEq/L) and hyperkalemia (potassium 5.2 mEq/L). The patient was transferred to our institution for further evaluation and subspecialty management.

Upon admission, repeat laboratory evaluation confirmed worsening pancytopenia: WBC 2.5 × 10^3^ cells/uL, absolute neutrophil count (ANC) 1180 cells/uL, hemoglobin 9.4 g/dL, and platelet count 18 × 10^3^ cells/uL. Additional studies revealed a markedly elevated lactate dehydrogenase (LDH) of 4361 U/L, severe hyperferritinemia at 40,930 ng/mL, and hypofibrinogenemia at 103 mg/dL. The combination of a falling hemoglobin, markedly elevated LDH, and schistocytes on peripheral smear was consistent with an ongoing hemolytic process at this stage of the workup and contributed to the initial clinical suspicion for thrombotic thrombocytopenic purpura (TTP). Peripheral blood smear review demonstrated ovalocytes, teardrop cells (dacrocytes), echinocytes, and rare schistocytes (Figure 1). No circulating blasts were identified. Notably, the reticulocyte count was subsequently found to be inappropriately low at 0.3%, supporting ineffective erythropoiesis rather than active hemolysis. However, this result was not available when the initial differential diagnosis was formulated. Computed tomography angiography of the chest revealed small bilateral pleural and pericardial effusions without pulmonary embolism. Abdominal imaging identified possible splenic infarcts. Triglycerides were elevated, further raising concern for a systemic inflammatory process.

**FIGURE 1:**
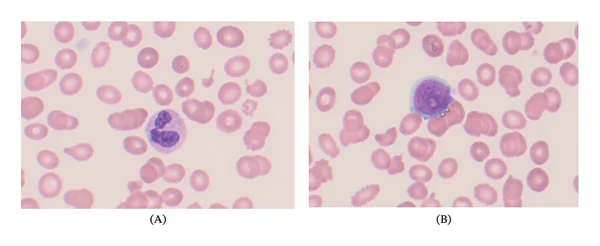
Representative peripheral blood smear (A) Teardrop cells (dacrocytes), ovalocytes, and echinocytes on a background of anisocytosis and poikilocytosis. A segmented neutrophil is visible centrally. (B) A circulating proerythroblast with prominent basophilic cytoplasm and a large round nucleus with open chromatin, surrounded by dysmorphic erythrocytes including rare schistocytes. These peripheral smear findings raised initial concern for a thrombotic microangiopathic process.

### 2.2. Differential Diagnosis and Initial Management

The combination of thrombocytopenia, elevated LDH, acute kidney injury, altered mental status, and peripheral smear findings including schistocytes, in combination with the laboratory picture described above suggestive of hemolysis, prompted high clinical suspicion for TTP. Hemophagocytic lymphohistiocytosis (HLH) was also considered given the extreme hyperferritinemia, hypofibrinogenemia, elevated triglycerides, and cytopenias, though this remained a diagnosis of exclusion. Disseminated intravascular coagulation (DIC) was entertained in light of the hypofibrinogenemia and critical illness. Separately, the pancytopenia, coagulopathy, and identification of rare circulating blasts (0.6%–1.5%) raised concern for acute promyelocytic leukemia (APML). Although the smear did not demonstrate classic hypergranular promyelocytes or blasts with Auer rods, the microgranular (hypogranular) variant of APML can present without readily identifiable Auer rods and may closely resemble other myeloid processes on routine smear review; in addition, the coagulopathy present in this patient is a hallmark of APML‐associated DIC but is not specific to it [11]. Given the high early mortality associated with untreated APML, this remained an important diagnosis to exclude and required urgent empiric intervention [12–14].

ADAMTS13 activity testing was sent to evaluate for TTP [15], and an urgent bone marrow biopsy was performed with expedited fluorescence in situ hybridization (FISH) for the t(15;17)(q22;q12) PML‐RARA translocation characteristic of APML. Pending results, empiric high‐dose corticosteroids and therapeutic plasma exchange (PLEX) were initiated for presumed acquired TTP [16, 17], and all‐trans retinoic acid (ATRA) was started concurrently given the concern for APML [11]. The patient initially demonstrated a partial clinical response, with improved sensorium, declining LDH and ferritin levels, stabilization of renal function, and correction of electrolyte abnormalities.

ADAMTS13 activity subsequently returned at 42%, which did not fulfill the diagnostic threshold for TTP (activity < 10%). Despite this result, PLEX was continued given the concurrent clinical improvement, and the inconclusive ADAMTS13 value was attributed to potential confounding by prior high‐dose corticosteroid administration, which may influence coagulation parameters and assay interpretation [18]. FISH for t(15; 17) was negative, and cytogenetic analysis did not identify the PML‐RARA rearrangement. ATRA was accordingly discontinued.

### 2.3. Clinical Deterioration and Definitive Diagnosis

Despite initial stabilization, the patient’s clinical course subsequently deteriorated. He developed progressive worsening of renal function culminating in anuria, recurrent leukopenia, and neutropenia, rising LDH, and worsening encephalopathy accompanied by severe abdominal pain. Repeat cross‐sectional abdominal imaging demonstrated marked inflammatory changes involving the cecum and ascending colon without pneumatosis intestinalis, consistent with neutropenic enterocolitis (typhlitis), as well as peripancreatic fat stranding raising concern for necrotizing pancreatitis (Figure 2). Surgical consultation was obtained; however, in the setting of advanced multiorgan dysfunction and poor projected outcomes, the patient and his care team made the shared decision to transition to comfort‐focused care.

**FIGURE 2:**
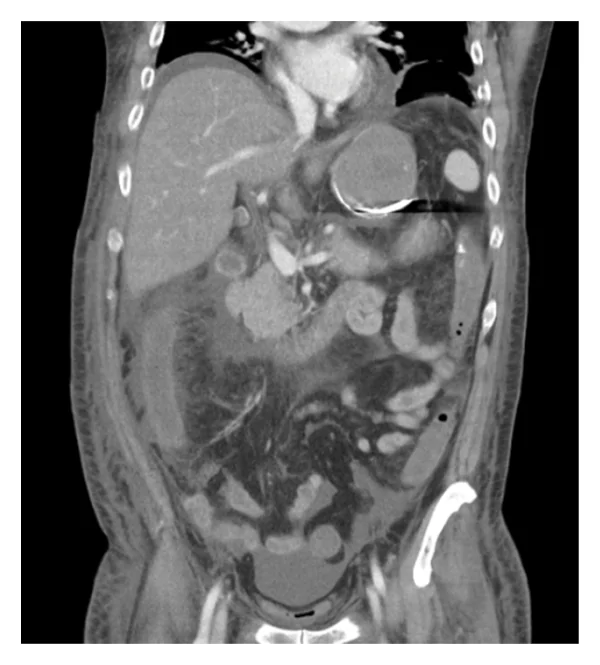
Contrast‐enhanced CT of the abdomen and pelvis (coronal reconstruction). Marked mural thickening and pericolonic inflammatory stranding involving the cecum and ascending colon (right lower quadrant), consistent with neutropenic enterocolitis (typhlitis), in the absence of pneumatosis intestinalis or pneumoperitoneum. Peripancreatic fat stranding (not shown) was also identified and raised concern for concurrent necrotizing pancreatitis.

The patient expired secondary to multiorgan failure prior to the initiation of definitive leukemia‐directed therapy. Final bone marrow biopsy and flow cytometry results, which had been pending from the time of initial admission, subsequently returned and demonstrated AML with greater than 90% erythroid blasts (Figures 3 and 4). Marrow cellularity was markedly elevated. The blast population was positive for CD33, CD71, CD43, dim/partial CD45, and p53, a pattern suggestive of erythroid lineage differentiation [19, 20]. CD71 is a transferrin receptor expressed broadly across proliferating and myeloid cell populations and is not specific to the erythroid lineage. More specific erythroid markers, including E‐cadherin and glycophorin A, were not performed. Similarly, CD117 and megakaryocytic markers such as CD61, CD42b, and von Willebrand factor were not assessed, which limited our ability to exclude a bipotential erythroid/megakaryocytic process. Despite these limitations, the molecular and cytogenetic findings provided additional evidence supporting the classification of this process as an aggressive, genetically complex myeloid neoplasm. Next‐generation sequencing (NGS) demonstrated JAK2 V617F (VAF 74%) and TP53 p.C238Y (VAF 66%) mutations, while cytogenetic analysis showed a triploid male karyotype with structural abnormalities involving 7p and 19p, including additional unidentified material at 7p21 and 19p13.3. These findings support TP53 pathway disruption in the setting of an antecedent JAK2‐mutated MPN and provide molecular context for the erythroid‐predominant phenotype [7, 8].

**FIGURE 3:**
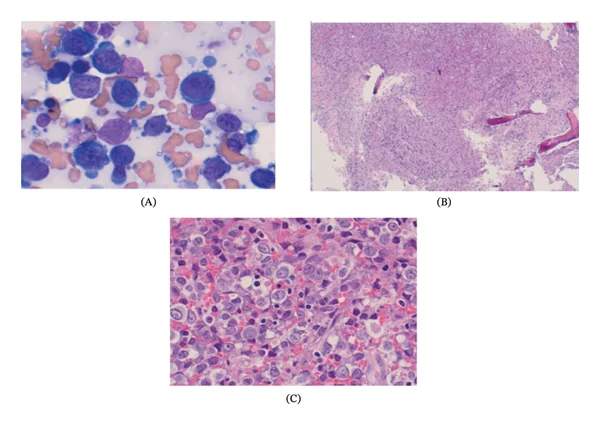
Bone marrow morphology. (A) Touch imprint preparation demonstrating a markedly cellular specimen with a predominance of large, immature cells with high nuclear‐to‐cytoplasmic ratios, dispersed chromatin, and prominent nucleoli, consistent with blast‐predominant marrow involvement. (B) Core biopsy at low power (× 4, hematoxylin and eosin) demonstrating markedly hypercellular marrow (> 90% cellularity) with effacement of normal architecture. (C) Core biopsy at high power (× 40, hematoxylin and eosin) demonstrating sheets of large, pleomorphic erythroid blasts with prominent nuclear irregularity, multinucleation, and mitotic figures, consistent with pure erythroid leukemia.

**FIGURE 4:**
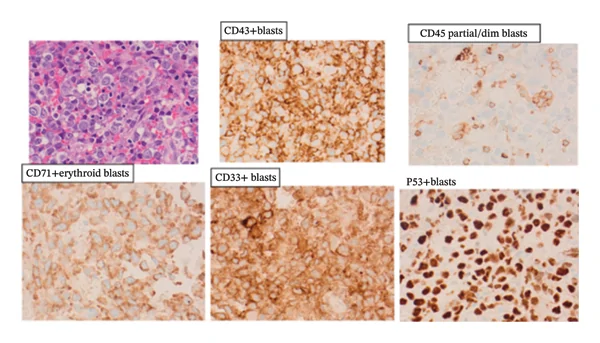
Immunohistochemical staining panel of bone marrow core biopsy. The blast population demonstrates diffuse positivity for CD43 and CD71 suggestive of erythroid differentiation. CD33 expression is present, consistent with myeloid antigen coexpression. CD45 shows dim/partial staining, in keeping with the erythroid lineage phenotype. p53 is strongly and diffusely positive, indicating TP53 protein overexpression consistent with a loss‐of‐function TP53 mutation (confirmed by next‐generation sequencing as TP53 p.C238Y, VAF 66%). The upper left panel shows hematoxylin and eosin staining at high power for morphologic reference.

### 2.4. Diagnostic Challenges Mimicry of Thrombotic Microangiopathy and APML

This case illustrates the diagnostic complexity that can arise when erythroid‐predominant AML presents with clinical and laboratory features that closely resemble other hematologic emergencies. At presentation, the co‐occurrence of a falling hemoglobin, markedly elevated LDH, and schistocytosis was interpreted as evidence of an active hemolytic process, and it was this constellation, together with thrombocytopenia and renal and neurologic involvement, that drove the initial suspicion for TTP. The reticulocyte count subsequently returned inappropriately low at 0.3%, favoring ineffective erythropoiesis over active hemolysis. However, this result was not available during the initial evaluation and did not inform the early diagnostic or management approach. Thus, the initial presentation was evaluated based on the available clinical and laboratory data, with the underlying diagnosis becoming clearer only after subsequent testing. Given the high mortality associated with untreated TTP and the well‐established principle that empiric PLEX should not be delayed pending ADAMTS13 confirmation, initiation of therapeutic PLEX was clinically justified [15, 16]. Prior randomized controlled trial data support the superiority of PLEX over plasma infusion in TTP (40/51 versus 25/51 responses; *p* = 0.002; 11 versus 19 deaths; *p* = 0.036), with worse outcomes associated with treatment delays beyond 24 h [16, 17].

The subsequent ADAMTS13 result of 42% did not confirm acquired TTP, and the preceding administration of high‐dose corticosteroids may have confounded the assay. It is well recognized that corticosteroids can modulate von Willebrand factor–cleaving protease activity and coagulation parameters, potentially obscuring a true ADAMTS13 deficiency [18]. Malignancy‐associated TMA, a distinct entity from immune‐mediated TTP, may also produce peripheral smear findings indistinguishable from classical TMA in the setting of underlying hematologic malignancy, further complicating the diagnostic picture [1, 15]. This overlap has been previously described in case reports involving erythroid‐predominant leukemia, where circulating dysplastic and fragmented erythroid precursors may be misconstrued as schistocytes on routine smear review [1].

The concurrent concern for APML, given the coagulopathy and rare circulating blasts, appropriately prompted empiric ATRA administration despite the absence of classic hypergranular promyelocytes or Auer rods on the peripheral smear. Not all cases of APML present with the classic morphology; the microgranular (hypogranular) variant in particular can lack readily identifiable Auer rods and may be difficult to distinguish from other acute leukemias or reactive processes on routine smear review. Furthermore, the DIC‐type coagulopathy observed in this patient, characterized by hypofibrinogenemia, is a hallmark of APML but is not exclusive to it and can accompany a range of critical illnesses and other leukemias, including erythroid‐predominant AML itself. In time‐sensitive scenarios, the high early fatality rate of APML, estimated at 5% to 10% within the first week, justifies ATRA initiation prior to cytogenetic confirmation, particularly when morphology is atypical or equivocal [12]. Early ATRA therapy has been demonstrated to significantly reduce 30‐day mortality [13, 14], and combined ATRA plus arsenic trioxide achieves superior event‐free survival compared with ATRA plus conventional chemotherapy [11]. The absence of t(15;17) on FISH and cytogenetics ultimately excluded APML as a diagnosis in this case, and ATRA was appropriately discontinued once that result was available.

### 2.5. Transformation of PV to Erythroid‐Predominant, TP53‐Mutated AML

The transformation of PV to AML represents an established but relatively uncommon disease complication. Risk factors for leukemic transformation include older age, prior cytotoxic therapy (particularly alkylating agents and radioactive phosphorus), elevated WBC count, and the presence of high‐risk molecular features, most prominently TP53 mutations [5, 6]. The mechanism of transformation typically involves clonal evolution through the sequential acquisition of cooperating somatic mutations that confer a proliferative or survival advantage [6]. In this patient, the detection of a TP53 mutation (VAF 33.1%) as early as February 2022, at the time of a bone marrow biopsy demonstrating MF‐2 fibrosis, three years prior to the terminal presentation, supports a model of stepwise clonal progression from PV to myelofibrosis and ultimately to overt leukemia [5, 10].

Transformation of PV to an erythroid‐predominant AML phenotype, whether classified as PEL under WHO‐HAEM5 or AML with mutated TP53 under the ICC, is uncommon but increasingly recognized. Preclinical and clinical data suggest that concurrent JAK2 and TP53 alterations may contribute to this phenotype by promoting erythroid and megakaryocytic differentiation. In murine models, combined JAK2 activation and TP53 loss of function promote expansion of erythroid/megakaryocytic progenitors and leukemic transformation, while a recent clinical series found JAK1/2 mutations exclusively among AML with erythroid/megakaryocytic differentiation (AML‐EMD) cases with concurrent TP53 mutations. Together, these findings provide a biologic framework for the erythroid‐predominant phenotype observed in our patient [9]. In the present case, greater than 90% of marrow cells were erythroid blasts, with an immunophenotype positive for CD71 and CD43, along with CD33 and dim/partial CD45, a pattern suggestive of erythroid differentiation, although this was not confirmed by a full lineage‐specific panel [2, 19, 20]. The strong p53 expression by flow cytometry served as a surrogate marker for TP53 protein overexpression or loss‐of‐function mutation, further corroborated by the confirmed TP53 p.C238Y mutation at VAF 66% on NGS, indicative of a dominant, likely biallelic TP53 alteration in the leukemic clone [7, 8].

### 2.6. Prognostic Implications of TP53 Mutation

TP53 mutations are among the most prognostically adverse molecular alterations in AML, and their prevalence in erythroid‐predominant AML is disproportionately high compared with other AML subtypes [7, 8, 21]. TP53‐mutated AML is characterized by a high frequency of complex and nondiploid karyotypes, including triploid and near‐haploid configurations, clonal evolution with subclonal heterogeneity, and near‐universal resistance to conventional induction chemotherapy [21, 22]. In the present case, the triploid male karyotype with structural abnormalities at 7p21 and 19p13.3 is consistent with the cytogenomic landscape commonly observed in TP53‐mutated disease and likely reflects chromosomal instability driven by TP53 loss of function [7, 8].

Clinically, patients with TP53‐mutated AML typically achieve low rates of complete remission with standard induction regimens, and those who attain remission frequently relapse within months [21, 23]. Allogeneic hematopoietic stem cell transplantation (HSCT) remains the only potentially curative modality, but its feasibility is limited by patient performance status, comorbidity burden, and the inability to achieve adequate pretransplant disease control [24]. Alternative treatment strategies have been explored, including hypomethylating agents combined with venetoclax and ruxolitinib‐based combinations for MPN‐derived AML, although erythroid/megakaryocytic differentiation has itself been associated with BCL‐XL–mediated venetoclax resistance [25–28]. Investigational agents currently under evaluation in clinical trials include entrectinib combined with ASTX727 targeting TP53‐mutated AML biology, and RNA hybrid‐capture NGS approaches have identified NTRK fusions as potential therapeutic vulnerabilities in select hematologic malignancies [29, 30]. Machine learning and artificial intelligence algorithms are also under active investigation as tools to improve diagnostic accuracy and prognostication in acute leukemia [31].

## 3. Limitations

This report has several inherent limitations. As a single‐patient case report, the clinical observations are descriptive in nature and preclude causal inference or population‐level generalization. The immunophenotypic panel used to support erythroid lineage assignment (CD33, CD71, CD43, dim/partial CD45, and p53) is suggestive of erythroid differentiation; CD71 in particular is a nonspecific marker of proliferative activity rather than a lineage‐defining erythroid marker. Confirmatory markers such as E‐cadherin and glycophorin A, along with CD117 and megakaryocytic markers (CD61, CD42b, and von Willebrand factor) that would help evaluate for a bipotential erythroid/megakaryocytic process, were not obtained, as the patient expired before additional flow cytometric evaluation could be pursued and before remaining marrow material could be further characterized. This case highlights the importance of considering an underlying myeloid neoplasm when hemolysis, thrombocytopenia, and multiorgan dysfunction occur in a patient with a history of MPN, particularly when the clinical picture is not fully explained.

## 4. Conclusion

Erythroid‐predominant, TP53‐mutated AML arising from PV, classified as AML with TP53 mutation under the 2022 ICC or as PEL under WHO‐HAEM5 depending on the classification framework applied, is a rare, diagnostically elusive, and biologically aggressive malignancy [1, 2, 23]. Its clinical presentation may closely mimic thrombotic microangiopathy and, when accompanied by coagulopathy and atypical circulating blasts, may also raise concern for APML, leading to empiric treatment and diagnostic delay even when that treatment is ultimately appropriate given the stakes of an incorrect diagnosis [1, 12, 15, 16]. The co‐occurrence of a TP53 mutation, detectable in this case years prior to frank leukemic transformation, confers exceptional genomic instability, resistance to conventional therapy, and a dismal prognosis [7, 8, 21]. This case, together with recently reported series of JAK2/TP53 comutated erythroid and erythroid/megakaryocytic AML, highlights a potential pattern of leukemic transformation that may be difficult to recognize in patients with antecedent MPNs. Rapidly progressive cytopenias in this setting should prompt evaluation for acute leukemia, particularly when accompanied by increasing blasts, dysplastic erythroid or megakaryocytic features, or acquisition of high‐risk molecular abnormalities such as TP53. Importantly, morphology alone may not reliably establish the lineage of an evolving neoplasm. A comprehensive immunophenotypic evaluation should therefore be performed when erythroid or megakaryocytic differentiation is suspected. Finally, the diagnosis should be assigned using the specific classification system being applied, as the same case may be designated differently across classification frameworks, with important implications for prognostic interpretation [3–6, 9]. Early bone marrow biopsy with comprehensive flow cytometry, cytogenetics, and molecular profiling remains essential to establish a definitive diagnosis and initiate appropriate management without delay, and enrollment of eligible patients in clinical trials evaluating novel agents for TP53‐mutated AML should be prioritized [26, 30, 32].

**Nomenclature**
AML: Acute myeloid leukemia
APML: Acute promyelocytic leukemia
ATRA: All‐trans retinoic acid
BM: Bone marrow
DIC: Disseminated intravascular coagulation
FISH: Fluorescence in situ hybridization
HLH: Hemophagocytic lymphohistiocytosis
HSCT: Hematopoietic stem cell transplantation
ICC: International Consensus Classification
LDH: Lactate dehydrogenase
LER: Leukoerythroblastic reaction
MDS: Myelodysplastic syndrome
MPN: Myeloproliferative neoplasm
NGS: Next‐generation sequencing
PEL: Pure erythroid leukemia
PLEX: Plasma exchange
PV: Polycythemia vera
TMA: Thrombotic microangiopathy
TTP: Thrombotic thrombocytopenic purpura
VAF: Variant allele fraction

## Author Contributions

Writing–original draft, writing–review and editing, and clinical care: Thalia Padilla Kelley, Lam Duc, Jean Sabile, and Ronan Swords.

## Funding

This research received no specific grant from any funding agency in the public, commercial, or not‐for‐profit sectors.

## Disclosure

All authors have read and agreed to the published version of the manuscript.

## Consent

No written consent has been obtained from the patient as there are no patient identifiable data included in this case report.

## Conflicts of Interest

The authors declare no conflicts of interest.
